# Deep-Learning-Based Automated Rotator Cuff Tear Screening in Three Planes of Shoulder MRI

**DOI:** 10.3390/diagnostics13203254

**Published:** 2023-10-19

**Authors:** Kyu-Chong Lee, Yongwon Cho, Kyung-Sik Ahn, Hyun-Joon Park, Young-Shin Kang, Sungshin Lee, Dongmin Kim, Chang Ho Kang

**Affiliations:** 1 Department of Radiology, Korea University Anam Hospital, Korea University College of Medicine, Seoul 02841, Republic of Koreamallecot@gmail.com (C.H.K.); 2 Advanced Medical Imaging Institute, Korea University College of Medicine, Seoul 02841, Republic of Korea; 3 AI Center, Korea University Anam Hospital, Seoul 02841, Republic of Korea; 4 Institute for Healthcare Service Innovation, College of Medicine, Korea University, Seoul 02841, Republic of Korea; hyunjoon21@naver.com (H.-J.P.); louiskang18@gmail.com (Y.-S.K.); 5 JLK Inc., Seoul 06141, Republic of Korea

**Keywords:** rotator cuff tear, magnetic resonance imaging, deep learning

## Abstract

This study aimed to develop a screening model for rotator cuff tear detection in all three planes of routine shoulder MRI using a deep neural network. A total of 794 shoulder MRI scans (374 men and 420 women; aged 59 ± 11 years) were utilized. Three musculoskeletal radiologists labeled the rotator cuff tear. The YOLO v8 rotator cuff tear detection model was then trained; training was performed with all imaging planes simultaneously and with axial, coronal, and sagittal images separately. The performances of the models were evaluated and compared using receiver operating curves and the area under the curve (AUC). The AUC was the highest when using all imaging planes (0.94; *p* < 0.05). Among a single imaging plane, the axial plane showed the best performance (AUC: 0.71), followed by the sagittal (AUC: 0.70) and coronal (AUC: 0.68) imaging planes. The sensitivity and accuracy were also the highest in the model with all-plane training (0.98 and 0.96, respectively). Thus, deep-learning-based automatic rotator cuff tear detection can be useful for detecting torn areas in various regions of the rotator cuff in all three imaging planes.

## 1. Introduction

The rotator cuff stabilizes the glenohumeral joint during movement by compressing the humeral head against the glenoid [1]. The rotator cuff comprises the supraspinatus, infraspinatus, teres minor, and subscapularis muscles. Rotator cuff tears are the most likely source of shoulder pain in adults [2,3]. The incidence of rotator cuff tears is increasing with the improving life expectancy and it may affect up to 20–40% of people according to the report [4]. Although the exact pathogenesis remains controversial, a combination of intrinsic and extrinsic factors is likely responsible for most rotator cuff tears. Arthroscopic rotator cuff repair has become the standard care for rotator cuff tears [5,6]. At times, distinguishing a rotator cuff tear from other conditions, such as adhesive capsulitis, solely through physical examinations can be challenging. Therefore, imaging modalities play crucial roles in diagnosing rotator cuff tears. Both magnetic resonance imaging (MRI) and ultrasonography (US) are the best noninvasive modalities for identifying and evaluating rotator cuff lesions [7,8]. MRI allows for the evaluation of entire cuff lesions with a sufficient field of view, while US provides a limited window for rotator cuff tendons and is largely dependent on the operator’s skill and experience. As rotator cuff tendons are curved structures surrounding the humeral head, a single imaging plane has limitations in evaluating the entire cuff lesions. Some lesions may be well visualized in the coronal plane and some may be visualized in the sagittal or axial plane. Due to the anatomical and pathological complexities, even experienced musculoskeletal radiologists require attention and time to interpret shoulder MRIs. In addition to increasing incidence, advancements in scanning techniques have reduced scan times, leading to an increased number of examinations within a limited timeframe, and resulting in a considerable increase in the number of MRIs that need to be read. Despite the increase in the number of shoulder MRI scans, there is an insufficient number of experienced musculoskeletal radiologists, both in terms of spatial distribution and availability over time, from a realistic perspective. On a positive note, the growing number of shoulder MRI examinations can provide a wealth of data for developing automated deep learning models for MRI interpretation.

With the advent of deep learning techniques, numerous models have been applied to screen and assist in labor-intensive radiological tasks in musculoskeletal imaging, such as bone age assessment in the hand or elbow, fracture detection in axial or peripheral skeletons, arthritis grading in knee or sacroiliac joints, muscle quality quantification, muscle and bone segmentation in various sites, and the clinical prediction of outcomes [9,10,11,12,13]. Most of these tasks are time-consuming processes and some of them may even be impossible for a human radiologist to conduct. In shoulder MRI, the diagnosis of rotator cuff tear and the quantification of rotator cuff muscle degeneration are common indications for applying deep learning techniques as well as imaging time acceleration [14,15,16,17,18,19,20]. Shoulder MRI typically consists of over a hundred images from various sequences and imaging planes, which takes considerable time for interpretation. One of the primary roles of shoulder MRI is to screen for rotator cuff tears, and several previous studies have utilized deep learning techniques for rotator cuff tear detection in shoulder MRI [21,22,23,24,25]. Despite the good performances of reported studies, they have limitations in terms of the input data and labeling methods that can be applied in clinical practice. They used only coronal images or nonfat-suppressed images or classified them based on operational records, and did not consider subscapularis and infraspinatus tears. Because a rotator cuff tear can be obscured in a single imaging plane according to its location and size, evaluations in all planes are required.

This study aimed to develop and validate a screening model for detecting a rotator cuff tear in all three planes of routine shoulder MRI using a deep neural network (DNN).

## 2. Materials and Methods

This study was approved by the Institutional Review Board of Korea University Anam Hospital. Shoulder MRI scans were conducted between January 2010 and September 2019. All shoulder MRIs were performed using 3-Tesla machines, including Magnetom TrioTrim, Skyra, and Prisma (Siemens, Erlangen, Germany), as well as Achieva (Philips, Best, The Netherlands). The shoulder MRIs were conducted with a dedicated shoulder coil, with patients in the supine position and their shoulder joints neutrally positioned, with palms facing upward. These scans included at least one fat-suppressed axial, coronal, and sagittal imaging plane, with the imaging planes set to be orthogonal to the glenohumeral joint. The exclusion criteria comprised individuals under 20 years of age, contrast-enhanced examinations, arthrograpic examinations, postoperative images, and poor image quality due to factors such as motion artifacts and improper shoulder positioning. To ensure the highest standards of image quality, two board-certified musculoskeletal radiologists, each with more than 3 years of experience, assessed the appropriateness of each image. This assessment was based on both the radiologic reports and, on occasion, the images themselves. All images were stored in the Digital Imaging and Communications in Medicine (DICOM) format, which is a standard format for medical images, and they underwent a thorough anonymization process to protect patient privacy.

### 2.1. Image Labeling

**Figure 1:**
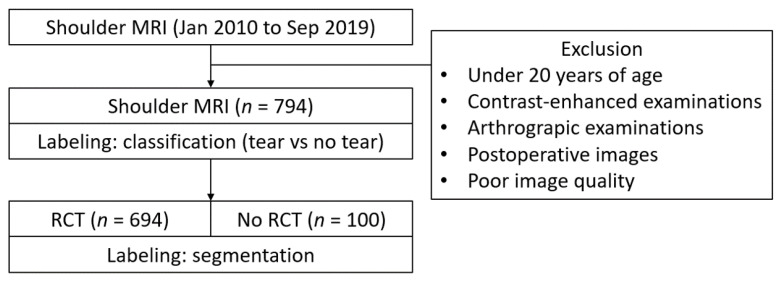
Flowchart of the study (RCT: rotator cuff tear).

Three board-certified musculoskeletal radiologists categorized the images as either “tear” or “no tear”, with evident full or partial fiber disruption of the tendon categorized as a “tear” and a normal tendon fiber or simple signal change of the tendon without fiber disruption regarded as “no tear”. All rotator cuff tears located in the supraspinatus, infraspinatus, teres minor, and subscapularis were meticulously examined in all axial, coronal, and sagittal planes of the shoulder MRI scans. Torn tendon spaces were segmented by trained researchers under the supervision of radiologists using AIX 2.0.2 (JLK Inc., Seoul, Republic of Korea). The flowchart of the methodology is demonstrated in Figure 1.

**Figure 2:**
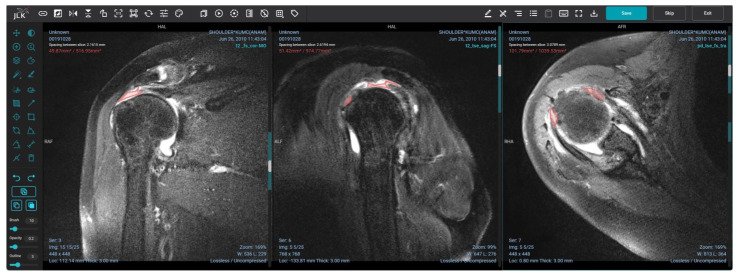
Segmentation of torn rotator cuff tendons on all three imaging planes. The segmentation is performed by drawing freeform lines (red) outlining all rotator cuff tears, including the supraspinatus, infraspinatus, and subscapularis, within all three imaging planes. Multiple areas of rotator cuff tears were segmented separately.

The segmentation process involved the creation of freeform lines outlining all rotator cuff tears, encompassing the supraspinatus, infraspinatus, and subscapularis, within all three imaging planes of fat-suppressed T2-weighted or proton density-weighted images (Figure 2). The cross-link function provided by the software assisted in identifying the corresponding point in the coronal image, which corresponds to the sagittal and axial images. In cases of multiple lesions, each rotator cuff tear was segmented separately. Subsequent to the segmentation procedure, rectangular patches were automatically generated, encompassing irregularly shaped torn segments. These patches were then utilized for the implementation of the model.

### 2.2. Model Implementation

**Figure 3:**
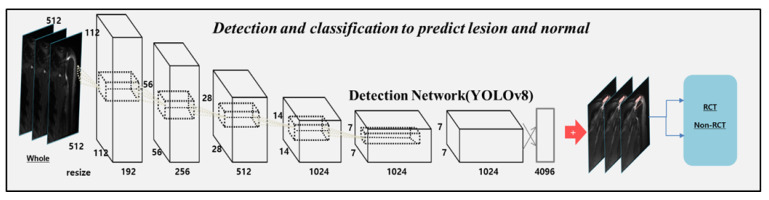
Network architecture of prediction model for rotator cuff tear.

The dataset was randomly divided into 70% for training, 10% for tuning, and 20% for the final evaluation. The algorithm was designed to detect and predict the rotator cuff tear. We used the original architecture of you only look once (YOLO) v8 [26,27] with a higher frequency of occlusion and small spatial sizes to improve the detection performance in shoulder MRI. This network was deeply fine-tuned and trained with regions of interest (ROIs) of the shoulder lesions and normal. After training, we examined the location and classification of lesions in the test sets. The primary purpose of YOLO v8 [26,27] involved partitioning each image using an S × S grid. The preceding iterations of YOLO v8, such as a novel neural network structure, incorporated both the Feature Pyramid Network (FPN) and Path Aggregation Network (PAN), along with an innovative annotation tool streamlining the labeling procedure. This annotation tool has multiple beneficial functionalities, including automated labeling, labeling shortcuts, and adaptable hotkeys. The amalgamation of these attributes simplified the process of annotating images for model training. The detection outcome should achieve a score of 0.5 or higher to emphasize the significance of both classification and detection [27]. All the datasets were resized to 512 × 512 pixels for training and inference. To enhance the performance of the model, the training datasets were preprocessed via histogram matching to align the histogram distributions across all images. In addition, all images underwent intensity normalization, which involved subtracting the mean and dividing it by the standard deviation. Resizing was achieved using third-order spline interpolation with linear interpolation. Furthermore, various image augmentation techniques were employed, including adjustments to the brightness, contrast, Gaussian noise, blur, inversion, and sharpness, and geometric modifications such as shifting, zooming, and rotation. These augmentations were employed to mitigate biases specific to scanners and bolster the resilience of neural networks against additional sources of variability unrelated to radiological categories. The tuning loss plateaued after an epoch, and the model with the lowest tuning loss was selected using the ADAM optimizer [28]. The structure of the model is illustrated in Figure 3.

These datasets were loaded onto a Graphics Processing Unit (GPU) devbox server with Ubuntu 20.04, CUDA 11.2, and cuDNN 11.1 (NVIDIA Corporation, Santa Clara, CA, USA), which is part of the NVIDIA deep learning software development kit (version 11.1). The GPU server contained four 48 GB A6000. We used an initial learning rate of 0.001 that decayed by a factor of 10 each time.

### 2.3. Statistical Analysis

We calculated the area under the curve (AUC) for the receiver operating characteristic (ROC) curve and accuracy using the pROC (version 1.10) package in R (version 1.42; R Foundation for Statistical Computing, Vienna, Austria). DeLong tests were performed to compare the AUC values of the eight classifier models using the pROC package in R version 1.42. Statistical significance was set at a two-sided *p* < 0.05.

## 3. Results

### 3.1. Subject Demographics

A total of 794 shoulder MRI scans were included (374 men and 420 women; aged 59 ± 11 years). Out of these, 100 subjects had no evidence of rotator cuff tear, while the remaining 694 had a rotator cuff tear. We extracted a total of 8756 image patches from patients with a confirmed rotator cuff tear and 2052 patches from those with no rotator cuff tears. The data distribution is presented in Table 1.

### 3.2. Performance of the Model

We first evaluated the performance of the model using the intersection of union (IOU) and confidence score (classification value of lesions) to evaluate the accuracy between the predicted bounding box and ground truth. If the IOU was over 0.5, the predicted lesions in test dataset were defined as correct. In addition, we used non-maximum suppression (NMS) to remove duplicate boxes for the inference of tears. To evaluate the detection performance based on YOLO v8, the cutoff threshold (0.2) was determined using the sensitivity and average false positives in the first algorithm.

**Figure 4:**
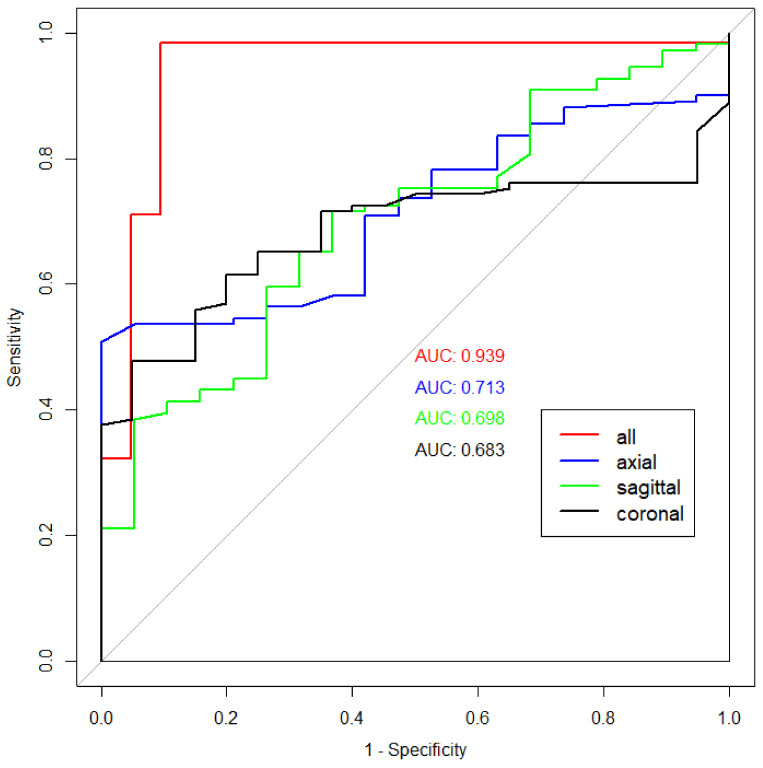
ROC curves for the model using all imaging planes (red) and using only axial (blue), sagittal (green), and coronal (black) images.

The highest AUC was achieved when all imaging planes were used (0.94), and this difference was statistically significant when compared to each individual imaging plane (*p* = 0.0002, 0.00006, and 0.00002, respectively). Sensitivity, precision, and accuracy were also the highest in the model with all-plane training. As a single imaging plane, the axial plane showed the highest AUC (0.71), followed by the sagittal (0.70) and axial (0.68) planes. The highest accuracy was achieved when using all imaging planes (96%). Regarding accuracy with a single imaging plane, the sagittal plane showed the highest accuracy (70%), outperforming the axial and coronal planes (58% and 55%, respectively). The performance of the model is summarized in Table 2, and the ROC curves for the model using all imaging planes and each individual imaging plane are demonstrated in Figure 4.

## 4. Discussion

In this study, we developed a screening algorithm based on YOLO v8 [26,27] to predict rotator cuff tear in shoulder MRI using high-quality datasets confirmed by expert radiologists. We used whole MRI images as the input data and used patch images drawn by musculoskeletal radiologists to train and fine-tune our algorithms. The advantage of this network is that it can simultaneously predict rotator cuff tear at various locations. It is important to determine whether the detection ability of the algorithm is similar to that of the expert radiologists in a computer-aided detection and diagnosis system. To the best of our knowledge, this is the first study to screen rotator cuff tear at all locations in all imaging planes.

The use of AI, especially deep learning techniques, has been introduced in various fields of musculoskeletal imaging, including radiography, computed tomography (CT), MRI, and US. The integration of deep learning techniques into radiography has yielded promising outcomes. Studies have shown its capability for swift and precise bone age assessment in hand or elbow radiographs, fracture detection across diverse anatomical regions, and the grading of osteoarthritis in knee radiographs [9,10,11,29]. In shoulder imaging, Kim et al. suggested using the deep learning model for ruling out rotator cuff tear in a shoulder radiograph, which redefined the role of a conventional radiograph [30]. Lee et al. reported a deep-learning-based model for analyzing a rotator cuff tear using ultrasound imaging [31]. Studies on quantifying rotator cuff muscle quality using deep learning has primarily relied on CTs and MRIs and have shown promising results. [16,32]. These tasks are recognized as labor-intensive, time-consuming, and, in some cases, even impossible for radiologists to perform. In the context of shoulder MRI, it is understandable that the evaluation of rotator cuff tears presents another promising application for deep learning, especially considering its increasing number of examinations and the lack of experts [21,22,23,24,25].

Shoulder MRI is difficult to interpret even by clinicians because of the anatomic complexity of the shoulder joint with small curved tendons and ligament structures. All three planes should be examined carefully because a partial volume averaging effect can obscure the lesion when referring to only a single imaging plane [33,34]. Although several studies have applied deep learning techniques to interpret shoulder MRI to diagnose rotator cuff tears, there have been limitations owing to the quality of the input data regarding the imaging sequences, imaging planes, and reference standards [21,22,23,24,25]. Kim et al. [21] and Sezer et al. [22] proposed a model for classifying rotator cuff tears from MRI, but their models were trained using only coronal images. Shim et al. [23] reported a rotator cuff tear classification model using a 3D convolutional neural network using three plane images. However, the labeling was based on the arthroscopic finding and used the DeOrio and Cofield classification system [35], which usually evaluates supraspinatus tears. Yao et al. [24] proposed a deep learning model for detecting only supraspinatus tears on T2-weighted coronal images. The far anterior portion of the supraspinatus or the far posterior part of the infraspinatus is not orthogonally perpendicular to the coronal plane, resulting in an unclear delineation of rotator cuff tears in these locations in the coronal images. This phenomenon applies to other imaging planes and to other rotator cuff areas as well. Many previous studies focused only on the supraspinatus tendon or did not mention subscapularis tears, which might have been overlooked and sometimes described as “hidden lesions” [36]. Although the supraspinatus is the most common location of rotator cuff tears, the subscapularis tendon, which is best seen in the sagittal and axial planes, should be included in screening. Our model detects rotator cuff tears in all imaging planes and assists in the diagnosis of rotator cuff tears within the numerous images found in shoulder MRI. This capability is potentially valuable for both diagnosis and treatment planning.

In our model implementation, we utilized the YOLOv8 model. In the preliminary evaluation, we compared the DenseNet classification model with the YOLOv8 model. However, the performance of the DenseNet model (AUC: 0.93; accuracy: 0.90) was not superior to the YOLOv8 model in the validation set. Despite several limitations, such as a lower accuracy in detecting small targets and substantial computational power requirements for feature extraction, YOLO is a powerful object detection algorithm that can be applied in various fields, notably in medical applications encompassing radiology, oncology, and surgery [37]. By rapidly identifying and localizing lesions or anatomical structures, YOLO has significantly improved patient outcomes while reducing diagnosis and treatment times and enhancing the efficiency and accuracy of medical diagnoses and procedures [37]. Recently, a completely new repository, which includes YOLOv8, has been introduced for the YOLO model. This repository serves as an integrated framework for training object detection, instance segmentation, and image classification models. YOLOv8 is a recent addition to the YOLO series and stands out as an anchor-free model. Unlike previous versions that rely on anchor box offsets, YOLOv8 directly predicts the centers of objects, resulting in faster NMS speeds. The model provides outputs, including box coordinates, confidence scores, and class labels (lesions). Despite the known drawbacks of the YOLO model, the YOLOv8 model has been used in various medical image applications in the field of radiology. In studies involving radiography and MRI, these models have demonstrated high accuracy in detecting conditions such as osteochondritis dissecans in elbow radiographs, identifying foreign objects in chest radiographs, and detecting tumors in brain MRI scans [38,39,40].

In this study, the model that was trained with all imaging planes exhibited the best performance (AUC: 0.94), while the model that was trained with a single imaging plane demonstrated a relatively lower performance (AUC: 0.71–0.68). Sensitivity, precision, and accuracy were also the highest in the model with all-plane training. Although the variation in the number of training images could be a contributing factor, the distinct shapes of tears in different imaging planes might contribute to enhancing the model’s rotator cuff tear detection performance. Furthermore, despite the small difference, the axial plane displayed the highest performance among the single imaging planes. This finding is intriguing, as the coronal or sagittal plane is generally preferred for rotator cuff tear detection, given that supraspinatus tears are the most common and well visualized in the coronal or sagittal planes [41]. To interpret the results differently, it might be possible that axial images contain more information about rotator cuff tears than conventionally believed. Human readers tend to focus on specific imaging planes when the rotator cuff tear is evident; however, the deep learning model independently screens all images and assesses the presence of tears. This functionality will assist radiologists in the labor-intensive and time-consuming process of MRI interpretation. In addition, if it is possible to find AI-driven imaging biomarkers for rotator cuff tears in axial planes, it might be an additional value for deep learning research.

Our preliminary study had several limitations. Firstly, we only conducted an internal validation test. Since our dataset comprised routine MRI protocols from various machines and vendors, it exhibited a significant degree of heterogeneity. Nonetheless, external validation using shoulder MRIs from other machines or institutions with concrete reference standards by multiple readers is necessary to validate our results. Additionally, a reader study comparing the model with human experts might also be required. Secondly, while our methods demonstrated good performance in terms of the AUC (0.94), achieving an enhanced specificity score is crucial for clinical applications. These challenges can potentially be addressed through the utilization of larger datasets, diverse augmentations, and algorithm enhancements. Thirdly, we did not specify the anatomical location of the rotator cuff tear, such as whether it affected the supraspinatus, infraspinatus, or subscapularis. Our model was primarily designed to screen for rotator cuff tears in numerous shoulder MRI images, and as such, it did not include the nomination of anatomical locations in its labeling. However, for practical clinical use by general physicians and orthopedic surgeons, specifying the location in addition to detecting the lesion is essential. With the additional detailed labeling or application of an automated anatomic labeling algorithm [42], the next version of the model can provide information about the location and size of the rotator cuff tear. Finally, our model combined both full-thickness and partial-thickness tears under the rotator cuff tear category. Subclassifying tears into full-thickness and partial-thickness categories may be necessary, as clinical decision making can vary based on the tear thickness. To address these issues, further development involving a larger dataset and more detailed labeling that includes the class of tear thickness is warranted. Since different grading systems are applied to supraspinatus and subscapularis tears, it might be necessary to take a step-wise approach: initially screening for rotator cuff tears using our preliminary model and subsequently classifying the tear details including the location and thickness using secondary models.

## 5. Conclusions

Our deep-learning-based automatic rotator cuff tear screening model effectively aided in the detection of rotator cuff tears across all three image planes. With the increasing number of shoulder MRI scans and a growing demand for lesion detection support, a deep learning model can effectively assist in detecting rotator cuff tears.

## Figures and Tables

**Table 1:** The number of the study participants and image patches.

Subjects			Training	Tuning	Testing
No RCT (*n* = 100)	Number of Patches		1511	150	391
	Plane	Axial	566	51	152
		Coronal	362	37	86
		Sagittal	583	62	153
RCT (*n* = 694)	Number of Patches		6427	795	1534
	Plane	Axial	753	237	435
		Coronal	2415	289	547
		Sagittal	2233	269	552

RCT: rotator cuff tear.

**Table 2:** The performance of the rotator cuff tear detection model for shoulder MRI.

	AUC	Sensitivity	Specificity	Precision	Accuracy	F1 Score
ALL	0.94	98%	91%	98%	96%	97%
Axial	0.71	51%	100%	100%	58%	68%
Sagittal	0.70	72%	63%	92%	70%	81%
Coronal	0.68	48%	95%	98%	55%	64%

## Data Availability

The raw/processed MR image dataset analyzed in this study is not publicly available.
